# Metallic sealants increase flux and change selectivity in supported molten-salt membranes

**DOI:** 10.1039/d4re00449c

**Published:** 2024-12-03

**Authors:** Liam A. McNeil, Guannan Chen, Wenting Hu, Evangelos I. Papaioannou, Ian S. Metcalfe, Greg A. Mutch

**Affiliations:** a Materials, Concepts, and Reaction Engineering (MatCoRE) Group, School of Engineering, Newcastle University Newcastle upon Tyne NE1 7RU UK greg.mutch@newcastle.ac.uk

## Abstract

Metallic sealants are widely used with high-temperature membranes. Here we show that their use in supported molten-salt membranes results in order-of-magnitude differences in CO_2_ flux and introduces O_2_ co-permeation. The ‘short-circuiting’ effect they introduce has important implications for the design of future experiments, and the interpretation of past work.

## Abstract 2

Metallic sealants ‘short-circuit’ molten-carbonate membranes, providing an additional route for CO_2_ (and O_2_) permeation.

## Graphical abstract

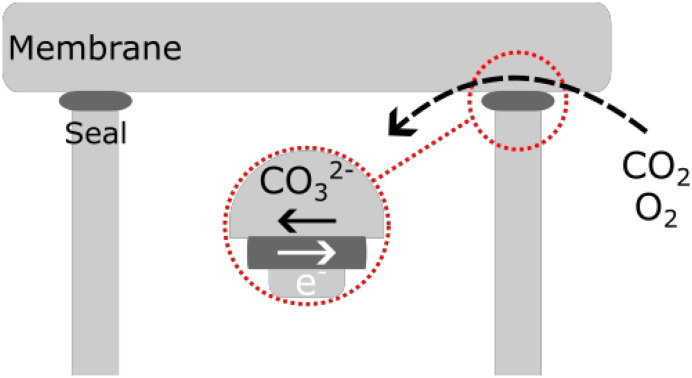

A key but often overlooked component of membrane modules and reactors is the sealant used to ensure that the fluid streams on the two sides of a membrane do not mix. However, to achieve this, the sealant must be in contact with both streams. Thus, the role of the sealant on flux and selectivity through a membrane should be considered. In high-temperature ceramic membranes, metallic sealants are frequently employed in the hot zone. Their use can, in principle, introduce poorly defined, transmembrane electronic conductivity.

Supported molten-salt membranes comprise a porous solid support, typically a metal oxide or metal, with molten salts infiltrated into the pore space *via* capillarity.^1^ Current literature suggests that they selectively permeate CO_2_ when they comprise molten carbonates supported in an oxygen-ion conductor *via*reaction 1, and that they co-permeate CO_2_ and O_2_ when an electronic conductor is employed *via*reaction 2.

1 CO_2(g)_ + O^2−^_(s)_ ⇌ CO_3_^2−^_(l)_

2
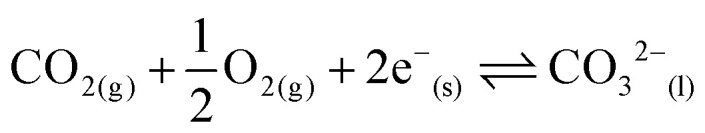


There are, however, contradictory results in the literature, which suggests there is unappreciated mechanistic nuance. The wide variety of support geometries and materials, salt compositions, and sealants used may be responsible for some of the contradictions as they introduce the potential for the superposition of different mechanisms in a single membrane (*e.g.*, reaction 2 introduced *via* the use of a metallic sealant to a membrane otherwise expected to follow reaction 1).

The lack of consensus around the CO_2_ permeation mechanism in arguably the simplest class of supported molten-carbonate membrane, *i.e.*, molten carbonates supported in a nominally inert material (*e.g.*, Al_2_O_3_), best highlights this issue. A variety of oxide-, hydroxide-, bicarbonate-, and carbonate-like species are posited as being stable in molten carbonates,^2–6^ which would in theory allow CO_2_ transport *via* a mechanism similar to reaction 1 in the molten phase alone. There have also been suggestions that electronic conductivity in molten carbonates (rather than in the support as in reaction 2) due to the formation of dissolved metal/cation pairs is responsible.^7^ Finally, the transport of neutral, dissolved CO_2_ (a solution–diffusion mechanism) is also possible, although comparing the physical and chemical (reactive) solubilities of CO_2_ in molten carbonates suggests such a mechanism likely does not contribute significantly.^1,8^

Despite inert supports providing lower CO_2_ fluxes than oxide ion- and electron-conducting supports, they provide very useful permeation and mechanistic data as they simplify the membrane by restricting permeation to the molten-carbonate salt alone.^9–13^ Moreover, with their low intrinsic CO_2_ fluxes, modifications to the membrane leading to increased fluxes can be easier to measure. For example, in our previous work, the growth of an electronically-conductive, transmembrane Ag structure within an Al_2_O_3_ support increased CO_2_ and O_2_ flux compared to the Al_2_O_3_ support alone.^12^ This was achieved by doping the molten-carbonate salt with Ag. Selectivity was also influenced, whereby O_2_ co-permeation (with CO_2_) occurred only in the membrane with Ag. Work on cermet-supported, molten-salt membranes has also shown that the incorporation of Ag into the membrane support provides high CO_2_ fluxes.^14,15^

Our previous work on Ag doping encouraged us to consider the potential impact of metallic sealants, as these are frequently employed with supported molten-salt membranes due to the high operating temperatures of these membranes (∼400–1000 °C). This has included Ag,^14–31^ and Au sealants,^32,33^ despite suggestions (but no experimental proof) that they might introduce poorly-defined, transmembrane electronic conductivity.^34,35^ An alternative approach is to avoid sealing in the hot zone, employing a cold-zone seal instead. However, at the lab-scale this has rarely been employed, as it can involve preparing larger and more complex dense membrane supports, followed by the introduction of porosity using *e.g.*, micro-scale subtractive manufacturing.^12,13,36^ Overall, this results in a much more expensive membrane and one which may not be suitable for real applications. However, as the sealant remains in the cold zone, the quality of the seal can be greatly improved which is important for interpreting permeation data and deriving mechanism.

Here we show that the use of metallic sealants with supported molten-salt membranes results in changes in flux and selectivity from feed-gas mixtures of importance for carbon dioxide separation. The use of a cold-zone-sealed Al_2_O_3_ support restricted permeation to the molten carbonate alone and demonstrated that molten carbonates selectively permeate only CO_2_ from both CO_2_/N_2_ and CO_2_/O_2_/N_2_ feed-gas mixtures. The use of both Au and Ag sealants in the hot zone with Al_2_O_3_ supports resulted in CO_2_ and O_2_ co-permeation from a CO_2_/O_2_/N_2_ feed-gas mixture, indicating a clear and significant impact on selectivity due to the transmembrane electronic conductivity introduced by the sealant. Furthermore, the metallic sealants increased CO_2_ flux significantly (∼1 × 10^−2^ ml min^−1^ cm^−2^ with Au, and ∼16 × 10^−2^ ml min^−1^ cm^−2^ with Ag). If supported molten-salt membranes are to progress towards scale-up, robust permeation and mechanistic data will be required; our results clearly demonstrate that consideration must be given to the sealants used.

## Experimental

### Membrane fabrication

Two membrane support geometries were employed: pressed pellets with a random porous architecture which required a hot-zone seal (Au or Ag), and tubular membranes with laser-drilled pores which required a cold-zone seal (O-ring and vacuum grease). These are hereafter referred to by the nature of the sealant, *i.e.*, as hot- or cold-zone-sealed supports (before carbonate infiltration) and hot- or cold-zone-sealed membranes (following carbonate infiltration).

To produce the hot-zone-sealed supports, Al_2_O_3_ powder (Alpha Aesar, ACS, >99.5%) was mixed with a 10 wt% PVA binder in a 1 ml binder : 1.5 g Al_2_O_3_ ratio. 1 g of this mixture was uniaxially pressed at 3 tonnes to form ∼1.75 mm thick, ∼20 mm diameter pellets using a hydraulic press. Pellets were sintered at 1200 °C for 5 h at a ramp rate of 2 °C min^−1^, before being sealed to Al_2_O_3_ tubes with two open ends (∼200 mm length, 12 mm outer diameter, 9 mm inner diameter) using commercial hot-zone sealants (Au and Ag pastes from Fuel Cell Materials). The metallic pastes were applied to the rim of the Al_2_O_3_ tube, before the tubes were pressed on to the pellets from above. Further metallic paste was applied around the external circumference of the Al_2_O_3_ tube, and the entire assembly was held in place with a clamp for ∼1 h at room temperature for initial adhesion. Metallic sealants were set at 850 °C in air, employing a heating rate of 1 °C min^−1^. After holding the support and sealant at 850 °C for 1 h, they were cooled at a rate of 1 °C min^−1^ to room temperature.

For the cold-zone-sealed supports, Al_2_O_3_ tubes (∼200 mm length, 20 mm outer diameter, 15 mm inner diameter) with one closed end of thickness ∼500 μm were laser drilled to form ∼2000 parallel pores within the central ∼15 mm diameter of the closed end. These laser-drilled tubes have been described in detail previously.^12,13,36^ They do not require a hot-zone sealant.

To prepare the carbonate phase of the membranes, individual lithium, sodium, and potassium carbonate powders (Alpha Aesar, ACS, >99.5%) were dried at ∼300 °C for ∼24 h in air, before being mixed in a ∼43.5 : 31.5 : 25 (Li : Na : K) mol% ratio.

### Membrane reactor

To seal both the hot- and cold-zone-sealed supports to the membrane reactor, a rubber O-ring and high-quality vacuum greases were applied to the open end of the Al_2_O_3_ tubes (noting that the hot-zone-sealed supports have the additional metallic sealant between the pressed pellet and Al_2_O_3_ tube). The open end of the tubes was situated in the base of the membrane reactor. The membrane reactor has been described in detail previously.^12,13,36^

Infiltration of supports was achieved by pressing the eutectic carbonate mixture into pellets which were placed on the surface of the membrane supports (∼20 and 15 mm diameter pellets of mass ∼0.6 and 0.06 g were employed for the hot- and cold-zone-sealed supports respectively, *i.e.*, they were of the same diameter as the porous areas of the supports and of a mass required to occupy the volume of the pores). The supports with carbonate pellets were enclosed within a quartz tube and heated to 450 °C at 1 °C min^−1^ under a flow of 50 mol% CO_2_ in N_2_ supplied to both feed and permeate sides. They were held at 450 °C for ∼1 h to permit infiltration of the molten carbonate (*T*_m_ ≈ 400 °C), before the reactor was heated to the experimental temperature (650 °C) at 1 °C min^−1^. At the experimental temperature the permeate-side gas was switched to Ar and the feed gas was either 50 mol% CO_2_ in N_2_ or 50 mol% CO_2_, 25 mol% O_2_ in N_2_. Detection of N_2_ at the permeate-side outlet above any pre-existing background level indicated a leaking membrane, at which point the experiment was abandoned. Similarly, CO_2_ or O_2_ permeation was assumed when signals exceeded their background level in Ar. All flow rates were 50 ml min^−1^, controlled by Brooks Smart II mass flow controllers, and measured at NTP.

### CO_2_ and O_2_ flux measurement

During permeation, the permeate-side outlet gas composition was monitored using a quadrupole mass spectrometer (Hiden Analytical QGA). The mass spectrometer was calibrated using Ar (background), and ∼0.04 and 1 mol% CO_2_ mixtures. Mole fractions of permeated gases were converted to volumetric flux, *J*_*i*_ (ml min^−1^ cm^−2^) using eqn (1),

1 *J*_*i*_ = *y*_*i*_ × *Q* × 1/*A*

where *y*_*i*_ is the mole fraction of species *i* in the gas phase, *Q* is the volumetric flow rate of the permeate-side gas in ml min^−1^, and *A* is the permeate-side area in cm^2^.^11^ The area used for the hot-zone-sealed membranes was ∼0.4 cm^2^ (based on the ∼7 mm inner diameter remaining after sealant spreading). For the cold-zone-sealed membranes, the area used was ∼1.8 cm^2^ (based on the ∼15 mm inner diameter/laser-drilled area).

### Membrane characterisation

Scanning electron microscopy (SEM), performed using a Tescan Vega 3LMU instrument, was used to analyse a membrane following gas permeation measurements. Digital images were collected using a digital camera and digital microscope.

## Results and discussion

**Fig. 1:**
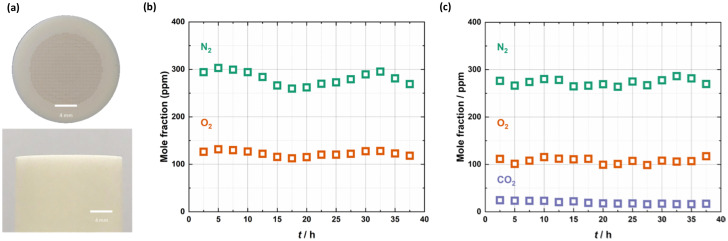
Leak testing with the cold-zone-sealed support and CO_2_ permeation in the cold-zone-sealed membrane. (a) Images of a cold-zone-sealed membrane. (b) Mole fraction of N_2_ and O_2_ in the Ar sweep-gas outlet, with a cold-zone sealed support without laser-drilled pores. (c) Mole fraction of N_2_, O_2_ and CO_2_ in the Ar sweep-gas outlet during a permeation experiment at 650 °C with a 50 mol% CO_2_, 25 mol% O_2_ in N_2_ feed gas.

Fig. 1 demonstrates that the cold-zone-sealed support and membrane possessed an exceptionally high-quality seal. First, a cold-zone-sealed support without laser-drilled pores was supplied with a 50 ml min^−1^ Ar sweep gas to determine the background mole fractions of N_2_ and O_2_ present due to *e.g.*, air leaks into the membrane reactor, mass spectrometer, and tubing/fittings used to connect the apparatus, and due to impurities in the Ar. N_2_ was present at ∼275 ppm and O_2_ at ∼125 ppm in the outlet of the sweep gas, suggesting that there were minimal leaks into the apparatus (Fig. 1b). Based on the ∼2000 : 1 (mole fraction in the air : mole fraction in the reactor) ratio for N_2_ and O_2_, this would suggest that the mole fraction of CO_2_ in the reactor as a result of air leaks would be on the order of 0.1 ppm.

Subsequently, the cold-zone-sealed membrane was supplied with a 50 mol% CO_2_, 25 mol% O_2_ in N_2_ feed gas and an Ar sweep gas at 650 °C. The lack of any significant change in the level of N_2_ indicated that despite the support having been laser drilled and infiltrated with molten carbonates, there were no measurable transmembrane leaks (N_2_ is not expected to permeate a molten-carbonate membrane) (Fig. 1c). The presence of CO_2_ at ∼20 ppm (two orders of magnitude higher mole fraction than that expected due to air leaks into the reactor) in the permeate-side outlet is due to selective permeation through the molten-carbonate salt (discussed below). We note that if the N_2_ and O_2_ background in Fig. 1b was subtracted from the permeation experiment in Fig. 1c, their mole fractions would become ∼0 ppm. This background subtraction methodology was applied in all experiments reported hereafter. Clearly, there is no measurable O_2_ flux, however, using the CO_2_ mole fraction from Fig. 1c, a low CO_2_ flux (<0.1 × 10^−3^ ml min^−1^ cm^−2^) can be calculated. Together, these results suggest that the molten-carbonate phase does not possess sufficient electronic conductivity to contribute significantly to CO_2_ permeation *via* a reaction like reaction 2, contrary to some previous proposals.^7^

**Fig. 2:**
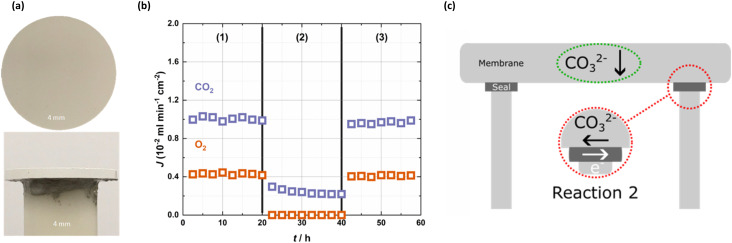
Short-circuiting due to the Au sealant. (a) Images of a hot-zone-sealed membrane. (b) CO_2_ and O_2_ fluxes as a function of feed gas at 650 °C in the Au, hot-zone-sealed membrane. An Ar sweep gas was used across all sections, with feed gas varying: (1) 50% CO_2_, 25% O_2_ in N_2_, (2) 50% CO_2_ in N_2_ and (3) 50% CO_2_, 25% O_2_ in N_2_. (c) Schematic of the short-circuiting effect due to metallic sealants, where reaction 2 is introduced *via* the metallic sealant.

As discussed above, we suspected that metallic sealants may introduce transmembrane electronic conductivity. Indeed, when hot-zone-sealed membranes were exposed to the same conditions (feed gases, sweep gas, and temperature) used with the cold-zone-sealed membrane, the results were quite different (Fig. 2). With a 50 mol% CO_2_, 25 mol% O_2_ in N_2_ feed gas ((1) and (3) in Fig. 2b), CO_2_ and O_2_ co-permeated the membrane sealed with Au, with a CO_2_ flux of up to ∼1 × 10^−2^ ml min^−1^ cm^−2^. We suggest that the introduction of transmembrane electronic conductivity to the membrane facilitated the co-permeation of CO_2_ and O_2_*via*reaction 2 by ‘short-circuiting’ the membrane (Fig. 2c).^34,35^ Moreover, the CO_2_ : O_2_ flux ratio in (1) and (3) of Fig. 2 is ∼2.5 : 1, but if the CO_2_ flux in (2) (note that the O_2_ flux is zero), where a 50 mol% CO_2_ in N_2_ feed gas was employed are subtracted, then the ratio is ∼2 : 1, consistent with reaction 2. This suggests that in (1) and (3), there is a superposition of reaction 2 (on top of the reaction responsible for permeation in (2)) due to the presence of O_2_ and transmembrane electronic conductivity *via* the Au sealant. Overall, Fig. 2 clearly demonstrates that the Au sealant ‘switches on’ selectivity for O_2_, and that when reaction 2 can occur due to the presence of O_2_ in the feed gas, CO_2_ flux is increased by ∼400% (comparing (1) and (3) to (2) in Fig. 2).

**Fig. 3:**
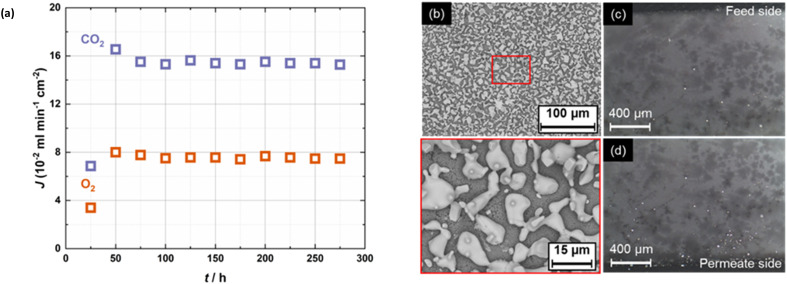
Significant short-circuiting due to the Ag sealant. (a) CO_2_ and O_2_ fluxes at 650 °C in the Ag, hot-zone-sealed membrane. An Ar sweep gas was used, with feed gas 50% CO_2_, 25% O_2_ in N_2_. (b) SEM images of the permeate side after use showing significant migration of the Ag sealant over the surface. (c and d) Images of the feed- and permeate-side cross-sections following the permeation experiment, showing migration of the Ag sealant into the bulk of the membrane.

The impact of metallic sealants was further evidenced by using the Ag sealant in place of the Au sealant (Fig. 3). In this case, again O_2_ co-permeation was observed with a 50 mol% CO_2_, 25 mol% O_2_ in N_2_ feed gas, but this time with significant migration of the Ag sealant across the surface of, and into the bulk, of the membrane (Fig. 3b–d). Although not shown here, migration of Au was not observed (this, and the mechanism of Ag migration is discussed in our previous work).^12^ Notably, the use of the Ag sealant resulted in the highest CO_2_ fluxes in this work (∼16 × 10^−2^ ml min^−1^ cm^−2^, an order of magnitude higher than with the Au sealant under identical conditions). The flux remained at this level for >200 h. Also, we note the CO_2_ : O_2_ flux ratio of ∼2 : 1, in line with reaction 2. In this case, with such high CO_2_ and O_2_ fluxes, the small contribution of any other permeation mechanism is concealed.

The very significant impact that the Ag sealant had on flux and selectivity is particularly concerning due to the aforementioned widespread use of Ag sealants in the supported molten-salt membrane literature.^14–31^Table 1 shows that a variety of Ag sealant forms (pastes, gaskets *etc.*) have been employed at the laboratory scale. This variety likely means that the effects we discuss (changes in flux and selectivity) will differ quantitatively between studies. Moreover, the sealant quantity and sealing area in laboratory-scale studies is relatively large when compared to membrane geometries used in industrial applications. Thus, one might expect quantitative differences here also. However, it is very important to note that this could result in performance (*e.g.*, flux) at the laboratory scale that cannot be realised upon scale up.

**Table 1:** Sealant materials and forms employed in the supported molten-salt membrane literature

Sealant material	Sealant form	Ref.
Au	Paste	32, 33
Ag	Paste	18–24, 27–30
	Gasket	14, 15, 31
	Not described	16, 17, 25, 26
Graphite	Gasket	6, 37
	Not described	17, 38–42

Finally, we note that whilst we have studied the impact of metallic seals, graphite seals (which have an electronic conductivity approximately one order of magnitude lower than metals) are also used with supported molten-salt membranes, and therefore may unintentionally introduce contributions to permeation *via*reaction 2 also.^6,17,37–42^ It is possible that oxide-ion conducting sealants may introduce reaction 1 contributions similarly.

## Conclusions

The influence that sealants have on flux and selectivity in membranes at the lab-scale should be carefully investigated as efforts towards the scale-up of membrane modules and reactors may otherwise encounter unforeseen difficulties. Here we have shown that metallic hot-zone sealants ‘switch on’ O_2_ selectivity in supported molten-salt membranes. This conclusion was supported by comparison to a cold-zone-sealed membrane, which showed no measurable O_2_ permeation under the same conditions. Furthermore, the metallic sealants significantly increased CO_2_ flux, with an order-of-magnitude difference observed between Au and Ag sealants. Going forwards, mechanistic interpretation, and comparisons of the performance between different membranes should consider the significant impact that sealant choice can have.

## Data availability

Data for this article are available at data.ncl at https://doi.org/10.25405/data.ncl.27045505.

## Author contributions

Funding acquisition (EIP, ISM, GAM); investigation (LM, GC, WH); supervision (WH, EIP, ISM, GAM); validation (WH); visualization (GAM); writing – original draft (GAM); writing – review & editing (WH, EIP, ISM, GAM).

## Conflicts of interest

There are no conflicts to declare.

## References

[cit1] Mutch G. A., Qu L., Triantafyllou G., Xing W., Fontaine M. L., Metcalfe I. S. (2019). J. Mater. Chem. A.

[cit2] Carper W. R., Wahlbeck P. G., Griffiths T. R. (2012). J. Phys. Chem. B.

[cit3] Xing W., Li Z., Peters T., Fontaine M. L., McCann M., Evans A., Norby T., Bredesen R. (2019). Sep. Purif. Technol..

[cit4] Cassir M., Moutiers G., Devynck J. (1993). J. Electrochem. Soc..

[cit5] Xing W., Peters T., Fontaine M. L., Evans A., Henriksen P. P., Norby T., Bredesen R. (2015). J. Membr. Sci..

[cit6] Cerón M. R., Lai L. S., Amiri A., Monte M., Katta S., Kelly J. C., Worsley M. A., Merrill M. D., Kim S., Campbell P. G. (2018). J. Membr. Sci..

[cit7] Näfe H. (2014). ECS J. Solid State Sci. Technol..

[cit8] Claes P., Moyaux D., Peeters D. (1999). Eur. J. Inorg. Chem..

[cit9] Wade J. L., Lee C., West A. C., Lackner K. S. (2011). J. Membr. Sci..

[cit10] Kazakli M., Mutch G. A., Qu L., Triantafyllou G., Metcalfe I. S. (2020). J. Membr. Sci..

[cit11] Kazakli M., Mutch G. A., Triantafyllou G., Gil A. G., Li T., Wang B., Bailey J. J., Brett D. J. L., Shearing P. R., Li K., Metcalfe I. (2021). J. Membr. Sci..

[cit12] McNeil L., Mutch G. A., Iacoviello F., Bailey J., Triantafyllou G., Neagu D., Miller T., Papaioannou E. I., Hu W., Brett D., Shearing P., Metcalfe I. S. (2020). Energy Environ. Sci..

[cit13] Metcalfe I. S., Mutch G. A., Papaioannou E. I., Tsochataridou S., Neagu D., Brett D. J. L., Iacoviello F., Miller T. S., Shearing P. R., Hunt P. A. (2024). Nat. Energy.

[cit14] Mendoza-Serrato C. G., López-Juárez R., Reyes-Montero A., Romero-Serrano J. A., Gómez-Yáñez C., Fabián-Anguiano J. A., Ortiz-Landeros J. (2022). Chem. Eng. Sci..

[cit15] Fabián-Anguiano J. A., Ramírez-Moreno M. J., Balmori-Ramírez H., Romero-Serrano J. A., Romero-Ibarra I. C., Ma X., Ortiz-Landeros J. (2021). J. Membr. Sci..

[cit16] Norton T. T., Lin Y. S. (2014). Solid State Ionics.

[cit17] Norton T. T., Lu B., Lin Y. S. (2014). J. Membr. Sci..

[cit18] Tong J., Zhang L., Han M., Huang K. (2015). J. Membr. Sci..

[cit19] Zhang L., Gong Y., Brinkman K. S., Wei T., Wang S., Huang K. (2014). J. Membr. Sci..

[cit20] Zhang P., Tong J., Huang K. (2017). J. Mater. Chem. A.

[cit21] Fang J., Tong J., Huang K. (2016). J. Membr. Sci..

[cit22] Tong J., Si F., Zhang L., Fang J. (2015). Chem. Commun..

[cit23] Zhang P., Tong J., Jee Y., Huang K. (2016). Chem. Commun..

[cit24] Zhang P., Tong J., Huang K. (2018). ACS Sustainable Chem. Eng..

[cit25] Fabian-Anguiano J. A., Ortega-Lugo R., Ramirez-Moreno M. J., Zeifert B., Gomez-Yanez C., Ortiz-Landeros J. (2021). Int. J. Appl. Ceram. Technol..

[cit26] Xu Z., Zheng Q., Wang S., Zhang Z., Liu Z., Zhang G., Jin W. (2021). J. Membr. Sci..

[cit27] Wang S., Tong J., Cui L., Zhang P., Zhou F. (2022). J. Membr. Sci..

[cit28] Zhang L., Tong J., Gong Y., Han M., Wang S., Huang K. (2014). J. Membr. Sci..

[cit29] Zhang L., Gong Y., Yaggie J., Wang S., Romito K., Huang K. (2014). J. Membr. Sci..

[cit30] Tong Z., Qiao X., Hou L., Tong J., Zhang P. (2024). ACS Sustainable Chem. Eng..

[cit31] Gili A., Bischoff B., Simon U., Schmidt F., Kober D., Görke O., Bekheet M. F., Gurlo A. (2019). Membranes.

[cit32] Zhang G., Papaioannou E. I., Metcalfe I. S. (2015). Energy Environ. Sci..

[cit33] Grima L., Mutch G. A., Oliete P. B., Bucheli W., Merino R. I., Papaioannou E. I., Bailey J. J., Kok M. D., Brett D. J. L., Shearing P. R., Metcalfe I. S., Sanjuán M. L. (2021). J. Membr. Sci..

[cit34] Starykevich M., Jamale A., Yasakau K. A., Marques F. M. B. (2022). J. Membr. Sci..

[cit35] Jamale A., Starykevich M., Marques F. M. B. (2022). J. Membr. Sci..

[cit36] Tsochataridou S., Mutch G. A., Neagu D., Papaioannou E. I., Sanjuán M. L., Ray B., Merino R. I., Orera V. M., Metcalfe I. S. (2020). ACS Appl. Mater. Interfaces.

[cit37] Yang L., Ricote S., Lundin S. T. B., Way J. D. (2020). Ind. Eng. Chem. Res..

[cit38] Lu B., Lin Y. S. (2014). Ind. Eng. Chem. Res..

[cit39] Lu B., Lin Y. S. (2013). J. Membr. Sci..

[cit40] Li Y., Rui Z., Xia C., Anderson M., Lin Y. S. (2009). Catal. Today.

[cit41] Ovalle-Encinia O., Lin J. Y. S. (2022). J. Membr. Sci..

[cit42] Ovalle-Encinia O., Lin J. Y. S. (2022). Chem. Eng. J..

